# Calcium chloride diluted in ethanol 95% as female sterilizing agent: effect of transcutaneous delivery in rats

**DOI:** 10.1186/s12917-022-03498-9

**Published:** 2022-11-12

**Authors:** Etiele Maldonado Gomes, Endreo Alan Pail dos Santos, Maria Elisa Trost, Gabrielle Christine de Souza Campos, Roberto Thiesen, João Paulo da Exaltação Pascon, Ingrid Rios Lima Machado, Maria Ligia de Arruda Mestieri

**Affiliations:** 1 Department of Surgery, Medical and Imaging of Small Animals, Federal University of Pampa, Uruguaiana, Rio Grande do Sul Brazil; 2 Department of Pathology, Federal University of Pampa, Uruguaiana, Brazil; 3 Department of Anesthesiology, Federal University of Pampa, Uruguaiana, Brazil

**Keywords:** Chemical castration, Sterilisation, Ovary, Ultrasound

## Abstract

**Background:**

Different fertility control methods are investigated as a tool for population control of free-roaming animals. Chemical castration using calcium chloride has been widely studied over the years in males, but there are few studies related to its use in females. Therefore, we aimed to evaluate the local effects, as a potential chemosterilant, of two concentrations of calcium chloride diluted in 95% ethanol when administered by transcutaneous ultrasound-guided intraovarian injection in rats. In this study, 30 female Wistar rats were randomly divided into three treatment groups, which consisted of transcutaneous ultrasound-guided intraovarian injection of: 0.9% sodium chloride solution (GC); 10% calcium chloride diluted in 95% ethanol (G10); 20% calcium chloride diluted in 95% ethanol (G20). The animals were subdivided into two evaluation times, 15 days (*n* = 5 of each group) and 30 days (*n* = 5 of each group) after the intraovarian injection. The ovarian diameter was measured using ultrasound image prior and immediately after the injection and after the treatment period. Furthermore, animals’ clinical evaluation, estrous cycles assessment, macroscopic examination of the abdominal cavity and histological evaluation of the ovaries were performed.

**Results:**

Ovarian ultrasound measurement revealed changes (*p* < 0.05) between ovarian diameters before and immediately after the injection in all treatments. Three animals in G20 had a small focal skin lesion at the injection site that evolved to total healing. Extended and abnormal estrous cycles were observed in G10 and G20. At gross examination, adhesions and ovarian cysts were noticed in both groups, G10 and G20. Also, the histopathology analysis revealed changes in ovarian architecture and vessel congestion in G10 and G20, but ovarian tissue damage was greater in the ovaries treated with the highest concentration (G20).

**Conclusions:**

The results indicate that 20% calcium chloride diluted in 95% ethanol may be a potential agent for inducing sterilization in females and was possible to be minimally invasively delivered.

## Background

The control of free-roaming animals is a focus of great interest for public health, due to their potential sources of transmission of zoonoses and the welfare risk of these animals [1]. To prevent unwanted pregnancies, and consecutively the increase in the population of stray animals, spay or neutering it is highly recommended, being the elective gonadectomy one of most used techniques [2]. Conversely, surgical procedures have a difficult access to the population with limited resources [3].

Nevertheless, nonsurgical forms of contraception, such as chemicals agents, may offer a less costly alternative for inducing sterilization, and can be applied more widely in castration programs, compared to the conventional use of gonadectomy [4]. In this context, calcium chloride dihydrate (CaCl_2_) diluted in ethanol is described as a potential chemosterilant agent [5]. Previous studies in male dogs and cats show that a single intratesticular application of CaCl_2_ when diluted in ethanol, seems to be more effective, promoting long-term azoospermia due to its sclerosing activity in dogs [6].

To date, studies investigating chemosterilant methods have generally focused only on males, mainly due to the ease of administration to the tests [5]. However, it is believed that the reproductive control of females is as important or even more imperative to avoid new litters and the beginning of a new reproductive cycle. Probably, the difficulty in accessing the ovaries without surgical exposure [7] can be cited as the greatest difficulty in performing the technique in bitches and queens.

Only one study using CaCl_2_ diluted in 95% ethanol in ovaries was found in the extant literature. Although it was able to promote reduction in ovarian mass in *Bos indicus* heifers [8] many questions about the effect of this chemosterilant in ovaries remain unanswered. Therefore, the aims of this study are to assess whether CaCl_2_ concentrations (10 and 20%) diluted in 95% ethanol are capable of inducing tissue damage at the ovarian site and possible clinical changes induced by the drug. In addition, to also assess whether the chemosterilant can be applied using the ultrasound-guided ovarian injection technique in rats. Furthermore, it is believed that the results obtained in this study are of great importance for the development of future research aiming the chemical castration in females.

## Results

### Clinical evaluation

According to visual inspection, three animals in the G20 had small (< 1 cm) focal skin lesions at the injection site unilaterally. Initially these lesions had a necrotic surface appearance, but all evolved to total healing within 15 days of treatment. In addition, without apparent changes in the parameters evaluated, one female of G10 died 3 days after the procedure and was referred for evaluation. However, no apparent cause of death could be determined after necropsy. According to the results obtained through the *RGS*, 1/10 of the animals in the GC (30 minutes after the procedure) and in the G10 (1 h after the procedure), and 6/10 of the animals in the G20 needed a single application of tramadol (10 mg/kg, SC). In G20, animals showed signs of pain between the 30 minutes to 12 hours after the procedure. Animals were evaluated again 30 minutes after the first administration of tramadol and no signs of pain were observed. Also, during the 36 h of evaluation, none of the animals needed a second application of tramadol.

### Estrous cycle monitoring

There was no significant difference (*p* > 0.05) in the number of estrous cycles recorded between treatments in the animals that were euthanized at 15 or 30-days after injection (Table 1). However, although without significant difference, in the first 15 days the G20 had a lower number of cycles (2.4 ± 0.48) and fewer days in estrus (1.8 ± 1.16) when compared to G10 (cycles: 3.25 ± 0.82; estrus: 2.25 ± 1.92) and GC (cycles: 3.2 ± 0.74; estrus: 2.4 ± 1.85). In the animals evaluated for 30 days, G10 was the group that presented the lowest number of cycles (5 ± 1.54) when compared to GC (6.4 ± 0.48) and G20 (5.6 ± 0.8). On the other hand, G20 was also the group that had lower days in estrus (8.8 ± 1.83) when compared to G10 (9.6 ± 4.07) and GC (9.2 ± 1.46). Also, normal (9/29) and extended (14/29) estrous cycles were observed in all groups, but abnormal cycles were only recorded in groups G10 (3/10) and G20 (3/10).

**Table 1:** Assessment of the estrous cycle after ovarian injection of calcium chloride diluted in ethanol or saline solution. Data expressed in mean values ± standard deviation within each group. Proestrus, estrus and diestrus are expressed in days

Day of euthanasia	Group	Number of cycles	Proestrus	Estrus	Diestrus
	GC	3.2 ± 0.74	2.4 ± 1.85	4.2 ± 0.74	7.8 ± 2.2
15	G1	3.25 ± 0.82	2.25 ± 1.92	4.25 ± 1.29	7.75 ± 1.47
	G2	2.4 ± 0.48	1.8 ± 1.16	2.8 ± 0.97	10 ± 1.26
	GC	6.4 ± 0.48	4.6 ± 1.74	9.2 ± 1.46	14.8 ± 2.2
30	G1	5 ± 1.54	3 ± 1.58	9.6 ± 4.07	16 ± 3.94
	G2	5.6 ± 0.8	4.20 ± 2.28	8.8 ± 1.83	15 ± 2.28

*GC* control group (saline solution); G10 = 20% calcium chloride diluted in 95% ethanol group; G20 = 20% calcium chloride diluted in 95% ethanol group

### Ultrasound assessment

**Fig. 1:**
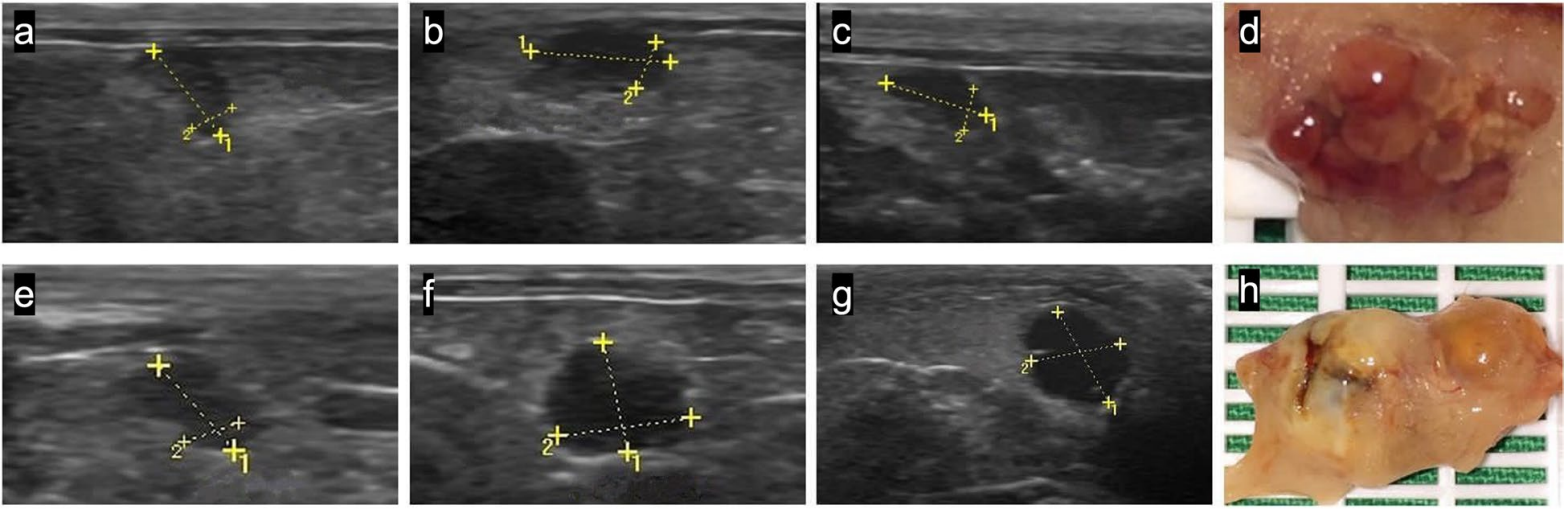
Comparison between ultrasound and macroscopic images of an ovary treated with saline solution (GC) and an ovary with a 20% CaCl_2_ injection (G20). Differences between prior to the application (0.40 cm) (**a**) and after the application of 150 μl of saline solution (0.51 cm) (**b**) were noticed. However, 15 days after the procedure (0.39 cm) (**c**) the ovary showed basal measurements. In the macroscopic image after euthanasia (**d**), the ovary showed normal structure and color. The G20 ovary showed an increase in its measurements when compared to prior to the application (0.32 cm) (**e**), after the application (0.53 cm) (**f**) and 30 days after the procedure (0.64 cm) (**g**). In gross inspection (**h**) it was possible to identify an ovarian cyst and adhesions between periovarian fat and ovary. *GC = control group (saline solution); G20 = 20% calcium chloride diluted in 95% ethanol group; cm = centimeters

Comparison between ovarian diameters prior to the application of intraovarian solutions (D1) and after the administration (D2) of all groups (Table 2) indicated an increase in organ size (*p* < 0.05) after the procedure (Fig. 1). No difference (*p* > 0.05) was observed between groups GC, G10, G20 measurements both in animals euthanized (D3) on the 15th and on the 30th. Also, means comparison between D1 and D3 measurements and comparison between D2 and D3 measurements were performed, but no significance was noticed (*p* > 0.05).

**Table 2:** Ovarian diameter calculated using ultrasound measurements in rats. Data expressed in mean values ± standard deviation within each group, in centimeters

		D1	D2	D3
	GC	0.38 ± 0.02	0.43 ± 0.02^*^	0.35 ± 0.01
15 days	G1	0.37 ± 0.02	0.41 ± 0.02^*^	0.38 ± 0.04
	G2	0.37 ± 0.02	0.43 ± 0.03^*^	0.38 ± 0.03
	GC	0.39 ± 0.01	0.44 ± 0.01^*^	0.37 ± 0.02
30 days	G1	0.37 ± 0.01	0.39 ± 0.01	0.40 ± 0.02
	G2	0.37 ± 0.02	0.44 ± 0.02^*^	0.43 ± 0.06
	General	0.38 ± 0.06	0.42 ± 0.07^*^	15d 0.37 ± 0.09^*^ 30d 0.40 ± 0.14

* Significative difference (*P* < 0.05). D1 = prior to the application of intraovarian solutions; D2 = after de administration of the solutions; D3 = euthanasia

### Gross examination

The main macroscopic findings are described in Table 3 and included the presence of adhesions and ovarian cysts (Fig. 1 h), which were only observed in the CaCl_2_ groups (G10 and G20). Also, since one animal was excluded from the study, the macroscopic evaluation was performed in 58 ovaries. In some of these, the adhesions involved tissues adjacent to the ovary such as uterine horn, abdominal wall, and kidneys. In one G10 animal, encapsulated calcified material adhered to the uterine horn was observed. Hemorrhagic ovaries with blackened focal areas were found in all groups.

**Table 3:** Distribution of post-mortem ovarian macroscopic and histological findings observed in all groups after intraovarian injection of calcium chloride or saline solution

		A + O	N/O	C.O.	H. O.	A.M.	M.C.	V.C.	SCORE	%
	GC	0	0	0	3	0	0	0	3/58	5.17
15 days	G1	3	3	1	2	2	3	2	16/56	28.57
	G2	4	7	1	2	4	2	3	23/61	37.7
	GC	0	0	0	3	0	0	0	3/70	4.28
30 days	G1	2	1	1	1	2	1	1	9/70	12.85
	G2	5	2	4	4	5	3	4	27/67	40.30

*A + O* Ovarian adhesions; *N/O* non-ovarian adhesions; *C.O.* Cystic ovary; *H.O.* Hemorrhagic ovary; *A.M.* area of mineralization; *M.C.* Morphological Changes; *V.C.* Vessel Congestion

### Histopathology

**Fig. 2:**
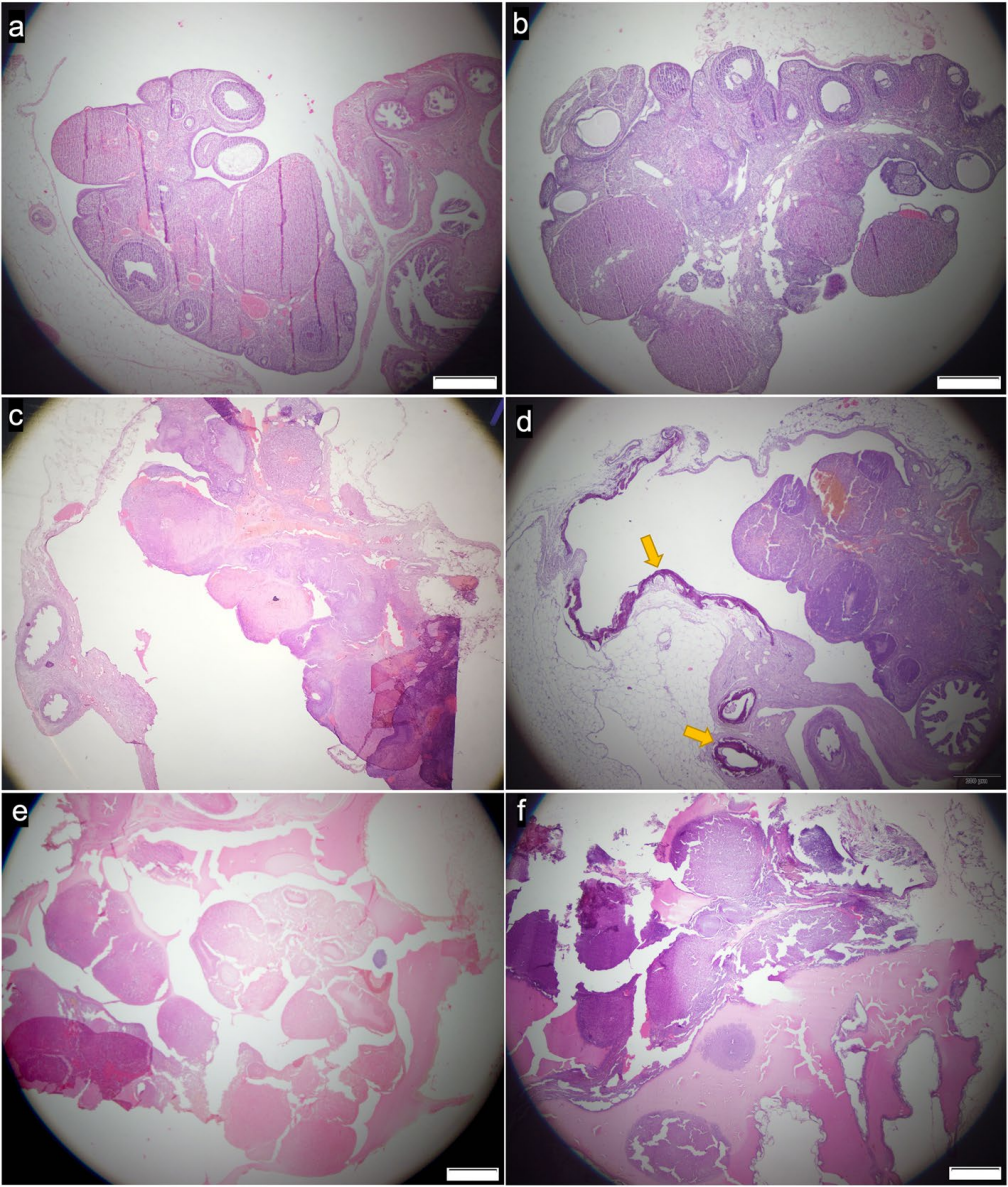
Histological images of ovarian morphology after intraovarian treatment with either saline (**a**, **b**), 10% (**c**, **d**) and 20% (**e**, **f**) CaCl_2_ solution in 95% ethanol. Ovaries were recovered either 15 (**a**, **c**, **e**; groups GC, G10 and G20, respectively) or 30 (**b**, **d**, **f**; groups GC, G10 and G20, respectively) after treatment. In images c, d, e and f it’s possible to notice morphological changes in ovarian parenchyma. Also, in image d, it could be noticed the presence of areas of mineralization (yellow arrows) in ovarian bursa and fallopian tube. *GC = control group (saline solution); G10 = 20% calcium chloride diluted in 95% ethanol group; G20 = 20% calcium chloride diluted in 95% ethanol group; cm = centimeters

**Fig. 3:**
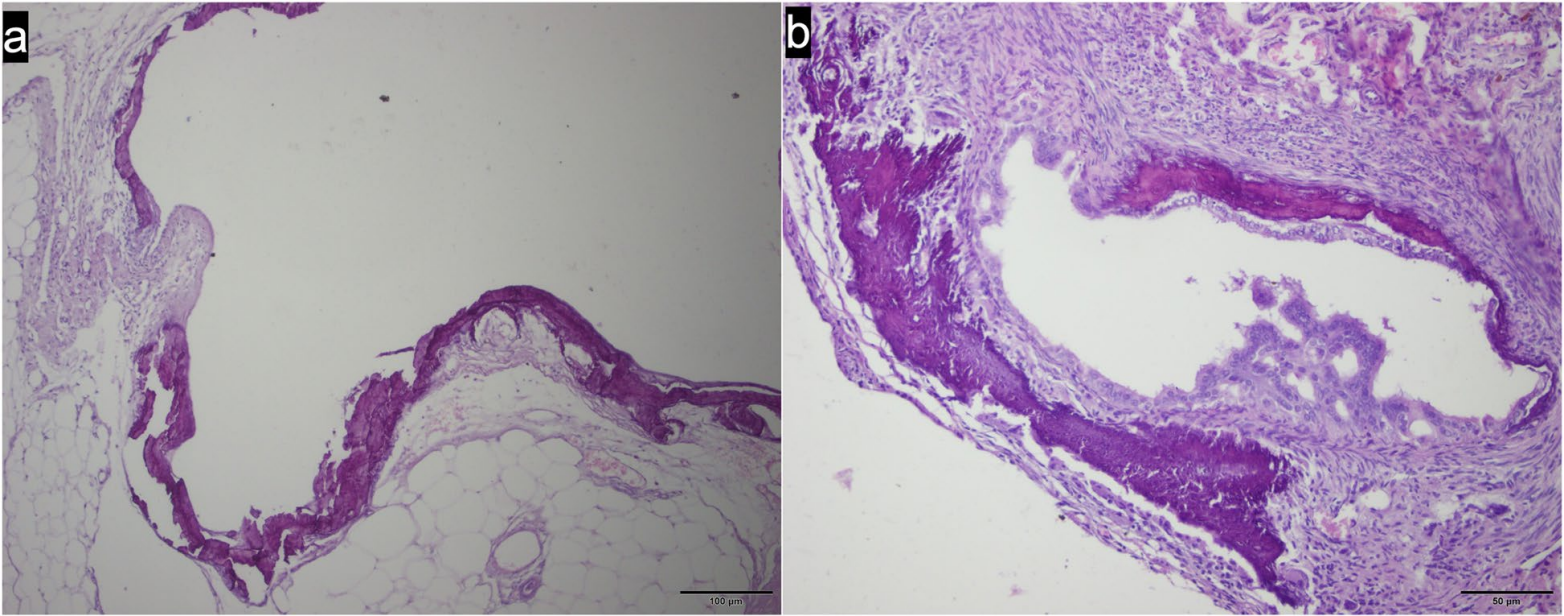
Histological image showing the presence of an area of mineralization in ovarian bursa (**a**) and in the fallopian tube in one animal of G10 (**b**)

The histological findings (Fig. 2) associated with the ovaries of each group are summarized in Table 3. In 8/58 ovaries, distributed among the groups, it was unable to perform the evaluation due to excessive periovarian fat and adhesions, which left no available ovarian tissue for analysis. Hemosiderin was frequently seen in all groups, but signs of blood vessel congestion were most observed in ovaries of G20 (7/16), and areas of mineralization could be noted also in fallopian tubes (Fig. 3) in G10 (4/18) and G20 (6/16). The presence of giant cells along with larger amounts of mononuclear inflammatory cells was observed only in the ovaries of animals in G10 and G20.

## Discussion

Both concentrations of CaCl_2_ (10 and 20%) diluted in 95% ethanol induced ovarian damage in rats after minimally invasive delivery, and the tissue damage induced by 20% CaCl_2_ was greater than that of 10% CaCl_2_. In histopathological analyses small areas of mineralization were observed, similar to findings observed in cattle ovaries after intraovarian administration of CaCl_2_ in alcohol [8]. In addition, other authors report that intratesticular use of CaCl_2_ promotes testicular morphological changes, including coagulative necrosis and fibrous tissue with architectural disruption, and focal calcium deposition [4, 9, 10]. In the present study, similar changes (Fig. 2) were mostly found in ovaries treated with CaCl_2_ 20% diluted in ethanol (G20).

The presence of giant cells in the ovaries treated with CaCl_2_ may be one of the inducing factors of apoptosis and consequent ovarian degeneration [11]. CaCl_2_ is known to induce necrosis [10], and apoptotic properties of CaCl_2_ have been previously reported [12], with both of which may occurring simultaneously. Another factor that may be related to giant cells is the production of free radicals [13], and this is directly related to induce tissue damage [14]. Nevertheless, as a limitation of this study, it was not possible to perform oxidative stress analysis of the ovaries. Further studies should evaluate the local activity of reactive oxygen species and antioxidant defenses.

Furthermore, it can be highlighted in this study that the highest injury scores (Table 3) in both assessment times belongs to G20, with the highest injury score (40.3%) observed 30 days after injection. These results also indicate that the ovarian tissue damage would be correlated in a concentration dependent manner, as observed in other previous studies in males [6, 9]. Similarly, the 10% CaCl_2_ concentration demonstrated important effects in the induction of ovarian damage in rats, which suggests greater studies to ascertain its potential in longer periods of time.

An important macroscopic finding in groups treated with CaCl_2_ was the intra-abdominal adhesions that might be generated as result of a chronic inflammatory process [15] and may contribute to clinical complications [16]. Proximity of the ovaries to the kidneys, as well as the presence of bowel loops and the spleen on the left side, require attention at the time of percutaneous ultrasound-guided injection. In this study, the largest number of animals with adhesions were in group G20, suggesting that the action of CaCl_2_ diluted in ethanol 95% occurs in dose-dependent manner, as has been reported in testicles [6]. However, no clinical complications and adherence involving bowel loops were observed.

The encapsulation of calcified material that was observed adjacent to uterine horns in rats treated with CaCl_2_ diluted in ethanol 95% may be related to the leakage of the content that may have occurred in some cases. CaCl_2_ could be identified as a foreign body and predisposes to the development of adhesions [17]. It was not possible to determine if these macroscopic findings occurred in consequence of the inflammatory process only or because of the leakage of injection content. As rats’ ovaries are encapsulated by a thin membrane [18] this may be not efficient in restraining the chemosterilant within the ovary*.* Differently from ovaries, testicles are surrounded by a thick and dense capsule [19], which possibly acts preventing leakage of the chemosterilant and limiting the inflammatory reaction to the organ. Other formulations of the chemosterilant should be tested in ovaries to optimize its retention and minimize adhesions.

Although three animals in G20 had a skin reaction, as previously reported [20], skin repair without major complication was observed within 15 days of treatment. Despite that, there was a loss of only one animal (1/30), although the cause of death was still unclear after necropsy. It is known that lower concentrations of CaCl_2_ tend to have less induction of side effects [6]. As this loss is from G10, the hypothesis that other intrinsic factors may have been the cause of death should also be considered as no death was observed in G20. In addition, although animals of all groups showed signs of discomfort after the procedure, a single application of tramadol was enough to provide analgesia and no signs of moderate or severe pain were observed 12 hours after the procedure according to the *RGS* results [21]. These results are in accordance with those previously described after intratesticular injection in dogs and cats, where even the control group with saline solution showed signs of discomfort, probably caused by the pressure of the injection [2, 9].

Animals from all groups showed changes in the estrous cycle, characterized by extended estrus and diestrus phases of the cycle. Prolongation of these phases may occur after exposure to stressors [22], animal aging [23] or xenobiotic treatment [24]. In this study, animals from all groups showed changes in the cycle, but since animal ages were homogeneous (90 days-old), stress would be the most likely cause of the extended cycles that were observed. However, as abnormal estrous cycles were only observed in animals in G10 and G20, this suggests that injection of CaCl_2_ was likely contributing to the expression of abnormal estrous cycles.

Ultrasound assessment showed that the injection was able to reach the ovary, providing an increase in the organ diameter immediately after the procedure. However, the measurements of ovarian diameter after treatments did not indicate any difference when compared to the previous diameter. This may be related to the induction of edema in the organ after the application of the drug, as previously described in males [25]. Studies suggest that for a detailed ovarian evaluation in rats, higher frequencies or ultrasound biomicroscopy [26] should be chosen, which should be considered for future research. However, the 13 MHz frequency was adequate to identify the ovary and perform the ultrasound-guided percutaneous injection technique.

Although the use of calcium chloride diluted in ethanol as a form of sterilization for domestic animals has been studied for decades, this is the first study to report its use in rats’ ovaries by ultrasound-guided technique. The findings in the present study revealed that CaCl_2_ diluted in ethanol causes ovarian lesions in rats in a dose-dependent manner, similar to what was previously observed in heifers [8]. Although it was not possible to identify ovarian atresia in any of the groups, CaCl_2_ diluted in ethanol delivered by a minimally invasive procedure showed good prospects for inducing damage to the ovary without leading to severe clinical complications. Future research should also evaluate if these ovarian lesions would be progressive, leading to total ovarian degeneration and consequently permanent sterilization, or if the tissue would be able to repair itself over time, and either not achieve sterilization or only temporarily disrupt fertility.

## Conclusion

CaCl_2_ diluted in 95% ethanol was able to be delivered in ovaries rats using a minimally invasive technique, with ultrasound guidance. Therefore, the technique demonstrated its potential use for minimally invasive ovarian procedures in female rats. The tissue lesions occurred in both CaCl_2_ concentrations, but ovarian damage was greater in higher concentrations of CaCl_2_ (20%) diluted in ethanol.

## Limitations

It is important to emphasize that, although it presents good perspectives, this study has limitations. Matting trials were not performed to evaluate the fertility status and determine the effectiveness of treatments to induce infertility. Also, calcium chloride intraovarian injection caused abdominal adhesions in some animals and, although it did not cause clinical complications for rats in this study, we do not know if in other species the clinical evolution would be the same. Finally, as a limitation of technique, the need to use ultrasound equipment can be cited as a limitation for the application use of the technique in different places.

## Methods

### Animals and experimental design

Thirty female Wistar rats were randomly divided in three groups (*n* = 10 animals/group). Animals were housed in plastic cages, given food and water ad libitum and maintained on a 12:12 h light:dark cycle in a controlled temperature environment. This study was approved by the Committee on the Ethics of Animal Use of the Federal University of Pampa (No. 020/2018). All methods were carried out in accordance with relevant guidelines and regulations. The study was carried out in compliance with ARRIVE guidelines.

Animals were acclimated for 2 weeks in the environment and with the researchers responsible for handling before administration of the intraovarian injections.

After this, each animal was subjected to general anaesthesia by inhalation of 4% isoflurane (Cristália, Itapira, Brazil) in a closed chamber. Once rats showed a loss of righting reflex, anaesthesia was maintained by 2% isoflurane inhalation using a nose-cone mask. Dorsolateral body hair of the flank region was shaved and scrubbed. An assistant located the ovary by digital palpation and restrained the kidney plus perirenal fat, so that the ovaries remained relatively stable for puncture. The ultrasound procedure was performed using a 13 MHz multifrequency linear transducer (GE Logiq P5, GE HealthCare®, São Paulo, Brazil). As a landmark for the ovary localization, the caudal pole of the kidney was identified by ultrasonography and a caudolateral and caudomedial scanning was performed for the ovary visualization. Subsequently, once the ovary was identified as a hypoechoic and ovoid structure, ovarian measurements were performed, and the needle (22-gauge) was percutaneously introduced until the ovary was reached, and then the solutions were administered.

Intraovarian percutaneous injection of 150 μl of solution was performed, guided per ultrasonography as previous described [27], in both ovaries, as follows: group GC (control) - saline solution; group G10 - calcium chloride 10% diluted in ethanol 95%; group G20 - calcium chloride 20% diluted in ethanol 95%. Previous practice on rats’ reproductive tracts showed that the volume of 150 μl of saline solution would be sufficient to fulfil the in rats of same age (90 days-old). In addition, the groups were subdivided according to the date of euthanasia, with five animals of each submitted to euthanasia at 15 or 30 days after the ovarian injection.

### Clinical evaluation

During the anesthetic recovery, animals were evaluated by two blind trained evaluators for signs of pain for 36 hours using the Rat Grimace Scale (RGS) to evaluate the analgesia requirement [21]. When the value obtained in the RGS was equal to or greater than 1 [21], the animal received analgesic rescue with tramadol 10 mg/kg by subcutaneous (SC) injection. In addition, the animals were evaluated daily to detect possible disorders resulting from the procedure. The parameters evaluated included signs of discomfort during abdominal palpation and visual inspection of the skin at the injection site.

### Estrous cycle

Vaginal cytological evaluation of all animals was performed daily until the euthanasia moment to determine the phase of the estrous cycle. The vaginal lavage was carried out with insertion of a pipette containing 100 μl of 0.9% saline solution in the vagina and aspirate the fluid immediately after. The aspirate was placed on a glass slide and analyzed under an optical microscope (40x) for cellular identification by a trained evaluator who was unaware of the treatment performed on the animals [28]. The phase of the estrous cycle was determined according to the proportion of cells identified. When performed in estrus, vaginal cytology presents nucleated and cornified cells [29], while in the proestrus there is a predominance of polynucleated cells, which may be dispersed or grouped [30, 31]. The presence of leukocytes, cornification of the cells and epithelial cells without specific cellular predominance is indicative that the animal is in the metaestrus [31]. The presence of leukocytes in greater number in the cytological evaluation is indicative that the animal is in the diestrus [28]. Animals’ cycles were also classified as normal (4–5 days of cycle), extended (3–4 consecutively days in estrus or 4–5 days in diestrus) or abnormal (> 4 days in continuous estrus or > 5 days in diestrus), as previous described [24].

### Ultrasound assessment

The ultrasound procedure was performed using a 13 MHz multifrequency linear transducer (GE Logiq P5, GE HealthCare®, São Paulo, Brazil). As a landmark for the ovary localization, the caudal pole of the kidney was identified by ultrasonography and a caudolateral and caudomedial scanning was performed for the ovary identification. Subsequently, once the ovary was identified as a hypoechoic and ovoid structure, ovarian measurements were performed in longitudinal plane (length and width) at three different moments: prior to the application of intraovarian solutions (D1), after the administration of the solutions (D2) and as soon as euthanasia was performed (D3). The ovarian diameter was estimated using the formula that was previously described [32]: (D = (length + width)/2).

### Euthanasia procedure

To perform the euthanasia, animals were anesthetized by intraperitoneal injection of ketamine (75 mg/kg; Cetamin, Syntec do Brasil Ltda., Santana da Parnaíba, Brazil) + xylazine (10 mg/kg; Kensol, Konig do Brasil Ltda., Mairinque, Brazil). Once rats showed loss of rightening reflex they were euthanized by intracardiac injection of 1 mL of 19,1% potassium chloride.

### Gross examination

The gross examination of abdominal cavity was performed immediately after euthanasia. Descriptive analysis included the presence of ovarian adhesions; non-ovarian adhesions; presence of cystic and/or hemorrhagic ovaries. These findings are presented in a scoring system, where each finding within the group represents a point. Therefore, the maximum score that each group can obtain is the total number of ovaries evaluated in the group versus the number of analyses performed.

### Histopathology

Left and right ovaries of each rat were collected for microscopic evaluation and preserved in 10% formalin for further processing. The material was sectioned at 3.5 μm thick in serial sections and two sections of each ovary, with 40 μm between them, were obtained. The slices were stained with hematoxylin and eosin and evaluated under optical microscope. Descriptive tissue analysis considered morphological changes, blood vessel congestion and areas of mineralization. Microscopic findings were also distributed in a scoring system, in the same manner as macroscopic findings.

### Statistical analysis

All statistical analyses were performed using IBM SPSS® Statistics software (version 20.0). Values that followed normal distribution (Shapiro Wilk test) were analyzed in One Way ANOVA and paired sample t-test. Data that did not follow the normality standard were submitted to Kruskal-Wallis and Wilcoxon Signed Rank Test. Mean values are reported together with the standard deviation (SD) and *p*-values < 0.05 were considered significant.

## Data Availability

The original contributions presented in the study are included in the article, further inquiries can be directed to the corresponding author.
